# Accelerating Biomolecular Modeling with AtomWorks and RF3

**DOI:** 10.1101/2025.08.14.670328

**Published:** 2025-08-15

**Authors:** Nathaniel Corley, Simon Mathis, Rohith Krishna, Magnus S. Bauer, Tuscan R. Thompson, Woody Ahern, Maxwell W. Kazman, Rafael I. Brent, Kieran Didi, Andrew Kubaney, Lilian McHugh, Arnav Nagle, Andrew Favor, Meghana Kshirsagar, Pascal Sturmfels, Yanjing Li, Jasper Butcher, Bo Qiang, Lars L. Schaaf, Raktim Mitra, Katelyn Campbell, Odin Zhang, Roni Weissman, Ian R. Humphreys, Qian Cong, Jonathan Funk, Shreyash Sonthalia, Pietro Liò, David Baker, Frank DiMaio

**Affiliations:** 1 Institute for Protein Design, University of Washington, Seattle, 98105, Washington, USA; 2 Department of Bioengineering, University of Washington, Seattle, 98105, Washington, USA; 3 Department of Biochemistry, University of Washington, Seattle, 98105, Washington, USA; 4 Department of Computer Science, University of Cambridge, Cambridge CB3 0FD, UK; 5 Graduate Program in Biological Physics, Structure and Design, University of Washington, Seattle, 98015, Washington, USA; 6 Paul G. Allen School of Computer Science and Engineering, University of Washington, Seattle, 98105, Washington, USA; 7 Graduate Program in Molecular Engineering, University of Washington, Seattle, WA 98105, USA; 8 Department of Computer Science, University of Oxford, Parks Rd, Oxford OX1 3QD, UK; 9 NVIDIA Corp., Santa Clara, USA; 10 Microsoft, AI for Good lab, Redmond, WA 98052, USA; 11 Cavendish Laboratory, Department of Physics, University of Cambridge, Cambridge CB3 0HE, UK; 12 Eugene McDermott Center for Human Growth and Development, University of Texas Southwestern Medical Center, Dallas, TX, USA; 13 Department of Biophysics, University of Texas Southwestern Medical Center, Dallas, TX, USA; 14 The Novo Nordisk Foundation Center for Biosustainability, Technical University of Denmark, DK-2800 Kgs. Lyngby, Denmark; 15 Department of Biotechnology and Biomedicine, Technical University of Denmark, DK-2800 Kgs. Lyngby, Denmark; 16 Howard Hughes Medical Institute, University of Washington, Seattle, WA 98105, USA

## Abstract

Deep learning methods trained on protein structure databases have revolutionized biomolecular structure prediction, but developing and training new models remains a considerable challenge. To facilitate the development of new models, we present AtomWorks: a broadly applicable data framework for developing state-of-the-art biomolecular foundation models spanning diverse tasks, including structure prediction, generative protein design, and fixed backbone sequence design. We use AtomWorks to train RosettaFold-3 (RF3), a structure prediction network capable of predicting arbitrary biomolecular complexes with an improved treatment of chirality that narrows the performance gap between closed-source AlphaFold3 (AF3) and existing open-source implementations. We expect that AtomWorks will accelerate the next generation of open-source biomolecular machine learning models and that RF3 will be broadly useful as a structure prediction tool. To this end, we release the AtomWorks framework (https://github.com/RosettaCommons/atomworks), together with curated training data, code and model weights for RF3 (https://github.com/RosettaCommons/modelforge) under a permissive BSD license.

## 1 Main

Deep learning has revolutionized the prediction of single chain proteins and their interactions, extending recently to include a wider set of biomolecules including DNA, RNA, and small molecules[1, 2, 3, 4, 5, 6, 7]. However, open-source efforts to develop and refine biomolecular machine learning models have been hamstrung by challenges that hinder progress. First, the heterogeneous data quality, annotations, and sources impose a considerable engineering overhead. Prospective researchers must spend months preparing data, optimizing processing, and battling edge cases within the Protein Data Bank (PDB)[8] before training any networks or, more commonly, opt to only handle a subset of the field’s complexity (e.g., protein-only models [9, 10, 11]). Second, the reliance on bespoke data pipelines for each network prevents generalization across use cases. Operations developed for one network cannot easily transfer to another, leading to duplication of code and effort across the research community [12, 13].

We reasoned that a modular, research-centric framework that enables rapid prototyping of protein structure prediction and design models would accelerate progress. We set out to develop such a framework within a high-performance codebase and utilize it to train open-source structure prediction and design methods.

## 2 Democratizing foundation modeling on biomolecular structure data

We describe below the tenets that guide development of AtomWorks, our generalized computational framework for biomolecular modeling. AtomWorks enables rapid prototyping and scalable training of biomolecular foundation models within one unified framework, emphasizing high-quality data handling, comprehensive documentation, and extensive testing to democratize access to biomolecular foundation modeling.

### AtomWorks prioritizes high-quality data.

Data quality is a key determinant of machine learning model performance. Within structural biology, the PDB is the primary experimental dataset, supplemented increasingly by model-derived distillation datasets as well [1, 5, 9]. These data sources, however, contain numerous edge cases that arise from the inherent heterogeneity of structural biology data. Correctly identifying and resolving these edge cases often requires expertise in biochemistry and structural biology. In AtomWorks, we begin by standardizing inputs with proper handling of these myriad data challenges; for example, identifying and removing leaving groups, correcting bond order after nucleophilic addition, fixing charges, parsing covalent geometries, imputing missing coordinates, and appropriate treatment of structures with multiple occupancies and ligands at symmetry centers. We find that our input processing approach translates to higher-quality derived downstream features. For example, AtomWorks reference conformers – generated through an empirical force field energy-minimization that is sensitive to erroneous charge and bond annotations – have lower energies than the reference conformers of another fully open-source model [7](fig. S2; median 67 vs 109 kJ/mol, as calculated by PoseBusters software suite [14]).

### AtomWorks maximizes research velocity by enabling rapid prototyping.

Current biomolecular modeling networks rely on independent data loading and featurization pipelines despite training procedures with many shared operations. To reduce duplicated efforts, we split subsequent data processing and featurization into modular components that operate on our common atom-level representation of structure. We further separate our logic into general-purpose operations that derive common features (e.g., load multiple sequence alignments, identify symmetric chains, generate reference conformers with chemoinformatics software such as RDKit [15]) and operations that translate the derived features into model-specific tensors. We find this approach both facilitates reuse of core building blocks across networks and reduces the complexity of adding new features (fig. S8). Previously, pipelines were single functions that incrementally converted input features into model tensors, discarding unneeded information along the way. Adding new features to such models involved introducing significant complexity to the code, leading to unmanageable data processing pipelines. Within the AtomWorks paradigm, the output of each step is not an opaque dictionary with model-specific tensors but instead an updated version of our atom-level structural representation (built on the open-source Biotite library’s AtomArray [16]). Operations within – and between – pipelines thus maintain a common vocabulary of inputs and outputs. Researchers need not contend with all existing features; they can focus on their specific experimental hypotheses, with knowledge that any modifications to the internal state (e.g., re-ordering or deleting atoms) will be appropriately handled downstream.

### AtomWorks enables the scalable training of biomolecular prediction and design models.

**Fig. 1:**
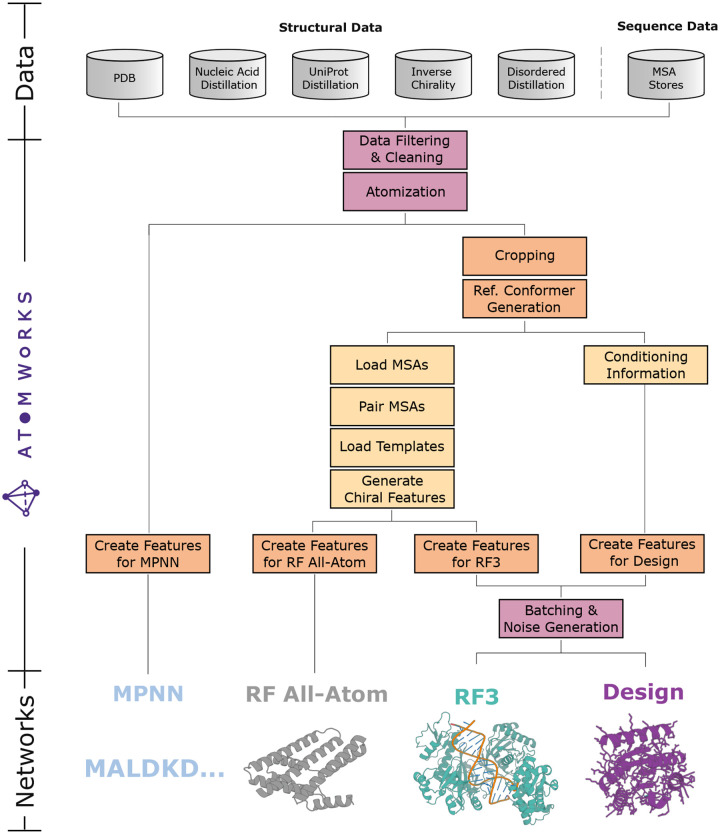
AtomWorks enables training of state-of-the-art models for structure prediction, generative protein design, and fixed-backbone sequence design within a single data framework. **Top**: AtomWorks can ingest (and RF3 is trained on) a diverse set of datasets including the Protein Data Bank (PDB), generated nucleic acid distillation sets, monomer distillation sets, PDB structures with inverted chirality, and PDB structures with extended disordered regions. **Middle**: Networks for all biomolecular modeling use cases can share common components within the AtomWorks framework. **Bottom**: Representations of outputs from various models trained with AtomWorks.

Building on this foundation, we implemented AtomWorks pipelines to train RF3, RF All-Atom (RFAA) [4], LigandMPNN [17], ProteinMPNN [18], and a to-be-published all-atom generative model (fig. 1). We find that the AtomWorks framework allows most code (>80%) to be shared across networks; researchers can repurpose existing components to rapidly test hypotheses, and improvements to common operations simultaneously benefit all methods. We contrast this approach to the prior paradigm where each model existed within a siloed codebase; component sharing was accomplished by copy-and-paste followed by modification to meet pipeline-specific needs. Indeed, the modular architecture of AtomWorks allows replacing 2,000+ lines of code in LigandMPNN with a 100-line declarative pipeline that borrows from operations originally written for RF3 and RFAA (fig. 1). Critically, our framework is also highly efficient. By relying on vectorized C operations and the pre-optimized Biotite library, we can parse from source structural files in milliseconds. This efficiency allows us to, for example, process a 6,000 token batch through our LigandMPNN pipeline within the time of a single forward/backward pass (0.6s).

### AtomWorks follows industry-grade testing and documentation practices.

We developed AtomWorks not as a point-in-time artifact but instead as a living library to be extended and improved. Currently, expert knowledge regarding how to handle the full complexity of biomolecular data is concentrated within a handful of academic labs and commercial entities. To address this disparity, we release alongside AtomWorks industry-grade tests for all operations (>85% coverage) and comprehensive documentation with worked examples illustrating how to develop pipelines for biomolecular modeling tasks. By focusing on usability, we facilitate the transfer of knowledge so that future researchers can more easily benefit from our foundation.

## 3 Training RF3

We use the AtomWorks framework to train RF3, an all-atom biomolecular structure prediction network competitive with leading open-source structure prediction networks. By including additional features at train-time – implicit chirality representations and atom-level geometric conditioning – we improve performance on tasks such as prediction of chiral ligands and fixed-backbone or fixed-conformer docking.

### RF3 simplifies dataset integration via AtomWorks.

As AtomWorks supports loading directly from raw crystallographic information files (CIF) through a unified processing pipeline, all that is needed to include additional training datasets are directories of predicted structures and, optionally, the corresponding MSAs. For RF3, we leverage this flexibility to introduce a number of novel distillation datasets. First, we incorporate a previously-reported distillation set of multi-domain proteins from AFDB truncated into pairs of interacting domains that mimic protein-protein interfaces [19, 20]. We also develop two new nucleic acid distillation datasets: a protein-nucleic acid complex distillation set and an RNA distillation set (with 27K examples and 10K examples, respectively; see supplemental methods) (fig. S1). Further, to address the issue of hallucinated secondary structure, we introduce a disordered distillation set that uses the Rosetta macromolecular modeling software [21] to generate structures with “extended” backbones for disordered regions (fig. 3b).

### RF3 accurately adheres to specified stereochemistry out-of-the-box, without inference-time guidance.

**Fig. 2:**
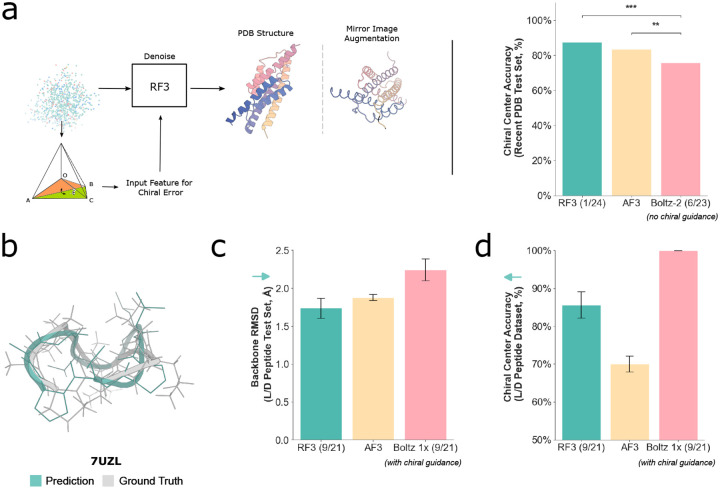
RF3 respects the chirality of the inputs. **A**: RF3 respects the chirality of small molecule inputs more than all other structure prediction models. We first cluster all small molecules in our test set by CCD code and average the percentage of correctly predicted centers within each cluster. Overall chiral center accuracy is computed by taking the mean across all clusters. Statistical significance was assessed using two-sample t-tests. RF3 and AF3 both significantly outperformed Boltz-2 (p < 0.001 and p = 0.002, respectively), while the difference between RF3 and AF3 was not significant (p = 0.064). Model training date cutoff indicated in parentheses. **B**: Comparison of predicted (teal) with ground-truth crystal structure (gray) for PDB ID 7UZL. In this example, 100% of the chiral centers are predicted with the correct chirality (including three D amino acids). **C**: On a test set of mixed chirality macrocycles from 2022, outside the training date cutoff of all models benchmarked, RF3 predicts the structures with a high degree of accuracy (1.74 mean backbone RMSD) **D**: In the mixed chirality test set, 85% of chiral centers are predicted correctly by RF3 vs. 70% by AF3. Boltz 1x structures were predicted with inference-time chiral guidance and thus are guaranteed to satisfy the input chirality.

**Fig. 3:**
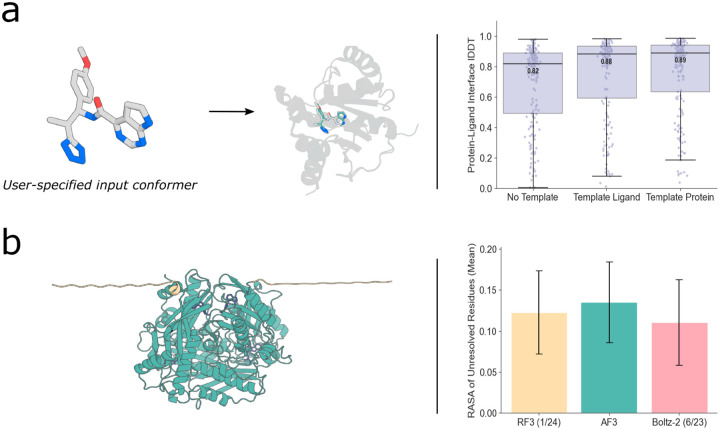
Novel capabilities of RF3. **A**. RF3 enables users to input desired conformers. (left) example of a user input conformer and prediction. (right) providing the ligand ground truth ligand conformer or the protein holo conformation improves accuracy. “Templating” in this case means providing all-by-all pairwise distances within the ligand or protein. **B**. RF3 is trained with a disorder distillation set. In contrast to AF3, which repredicted the entire PDB with AF2 to show examples of extended disordered regions, we chose to use the more compute-efficient Rosetta macromolecular modeling software to generate structures with “extended” backbones for disordered regions. In 2% of cases, the model is trained with PDB examples with extended disordered regions. (left) example of a prediction with a large unresolved region which is predicted in an extended conformation. (right) Analysis of the mean RASA over unresolved regions in our test set. Model training date cutoff indicated in parentheses.

To resolve the well-documented issue of incorrect chirality within diffusion-based structure prediction models, we represent stereochemistry by the sign of the angles formed by the atoms surrounding each chiral center. At each denoising step, we provide the diffusion model with a vector feature of the gradient of the error of the ideal angle (fig. S7, [4]). In addition, to encourage the model to respect the chiral features, we invert the chirality in 2% of PDB examples as a train-time data augmentation. We find that after making these improvements, the network predicts the correct chirality for 88% of ligand chiral centers within our test set, compared to 84% for AF3 and 76% for Boltz-2 without inference-time guidance (fig. 2a).

We further reasoned that the improved handling of chirality would enable RF3 to accurately predict mixed L/D peptides, a growing therapeutic class of molecules noted for their proteolytic stability and structural diversity. On a set of cyclic mixed L/D peptides from after the training date cutoff of all methods [22], we find that RF3 predicts 86% of chiral centers correctly (compared to 70% for AF3 and 100% for Boltz-1x, when using inference time guidance to enforce chirality; 2d). Moreover, the structures are highly accurate (mean 1.74Å backbone RMSD vs. 1.88 for AF3 and 2.24 for Boltz-1x; 2c). We hypothesize that inference-time modifications may shift the network outside the training distribution, which would explain why we find improved accuracy from representing chirality as a learned feature.

### RF3 enables flexible user control through arbitrary atom-level conditioning.

Users may specify distances between atoms that they want recapitulated in the output structure to enable incorporation of experimentally derived constraints, protein- or ligand-docking against a known protein structure, or protein folding around a specific ligand conformer. To measure the ability of the network to adhere to these constraints, we test the network on *holo* structure docking and protein folding around a rigid conformation of a ligand. When folding the protein around a rigid ligand, median protein-ligand interface accuracy increases from 0.821 to 0.882. Similarly, when providing distances constraining the *holo* structure, protein-ligand interface accuracy improves from 0.821 to 0.890 (fig. 2a). In all cases of templating the ligand, the model respects the given small molecule conformation (median ligand-only lDDT 0.991, fig. S6).

### RF3 narrows the performance gap between existing open-source structure prediction and AF3.

**Fig. 4:**
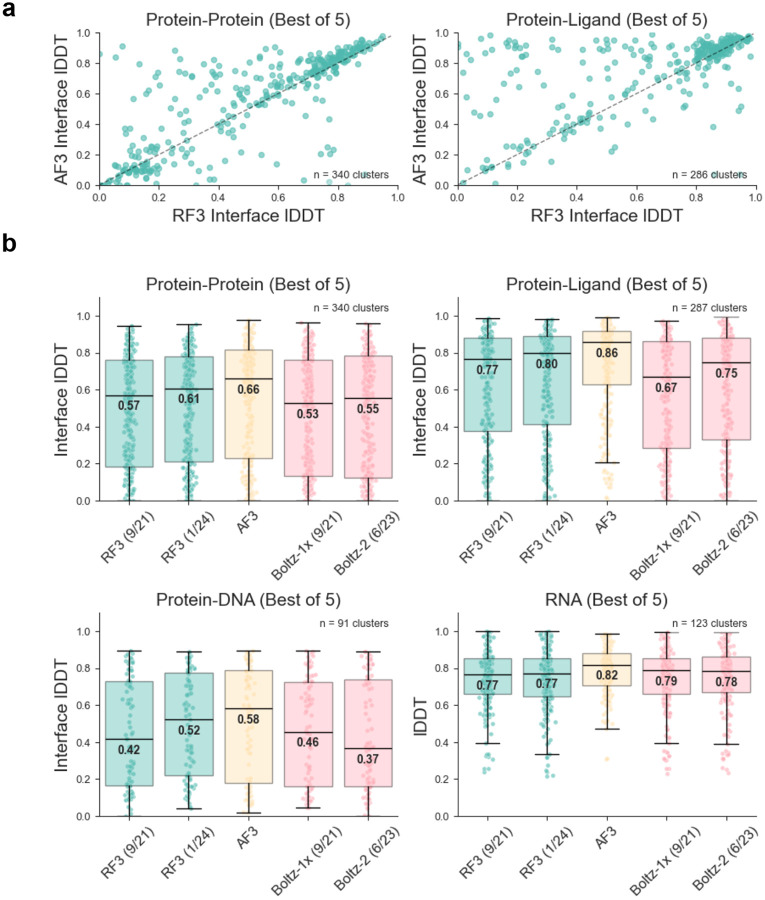
RF3 accurately predicts biomolecular interactions. **A**. Scatterplots comparing all-atom interface lDDT of RF3 and AF3 on protein-protein interactions and protein-ligand interactions. To reduce redundancy, the test dataset was clustered (by sequence homology 40% for polymers and CCD identity for non-polymers); each point represents a cluster mean. For all networks, we generate five structures from the same seed and take the sample that scores the highest on each metric (“Best of 5” approach). **B**. Boxplots showing accuracy of RF3, AF3 and Boltz on different structure modeling tasks. Two versions of RF3 are shown: one trained on structures released before September, 2021 and another trained on structures deposited before January, 2024. Each point in the boxplot is a mean value over a cluster of structures. Model training date cutoff indicated in parentheses.

Despite recent advances, open-source models continue to trail AF3 performance on most biologically relevant problems. In fig. 4, we compare the performance of RF3 to AF3 and another open source prediction network, Boltz [7]. All methods were run with a single seed, inference parameters to generate 5 diffusion predictions from one run of the trunk, and identical MSAs; for stability, we select the best-scoring sample for each metric (choosing a single structure with the confidence head shows similar trends, fig. S4). We find that the performance of RF3 is between the closed-source AF3 and open-source Boltz in almost all categories. When the training date cutoff is extended to 1/2024, we find RF3 performance increases across-the-board (0.571 vs. 0.607 median protein-protein interface lDDT, 0.766 vs 0.798 median protein-ligand interface lDDT, 0.415 vs 0.523 median protein-DNA interface lDDT and 0.765 vs. 0.772 median RNA only lDDT).

Antibody-antigen complex prediction represents a specific structure prediction use case with widespread applications within the pharmaceutical industry. When evaluated on a clustered, de-leaked test set of antibody-antigen complexes from the recent PDB, RF3 again performs between AF3 and the other methods (in this case, Boltz-2 and Chai-1): 33% of examples achieve DockQ > 0.23 for RF3, compared to 44% for AF3, 22% for Boltz-2, and 28% for Chai-1 (fig. S3).

## 4 Discussion

AtomWorks lowers the barrier-to-entry for training machine learning models on data from the Protein Data Bank and other structural biology databases, and RF3 sets a new standard for open-source models for prediction of the structures of protein-protein interfaces, protein-ligand interactions, and mixed L/D peptides. Using the AtomWorks modular data pipeline, scientists can develop new deep-learning networks without extensive software development experience. Our improved data processing directly translates to better generalization — when subset to cases with fewer than five similar ligands in the PDB, we find that reference conformer energies correlate with prediction accuracy between RF3 and Boltz (fig. S2). At the Institute for Protein Design, AtomWorks and RF3 have expanded both the number of developers who can contribute to deep learning projects and the code sharing between prediction and design efforts. We anticipate that these packages will be widely useful for new model development and to that end we release all data, code, and model weights for open use by the community.

## Supplementary Material

1

## References

[1] Jumper John, Evans Richard, Pritzel Alexander, Green Tim, Figurnov Michael, Tunyasuvunakool Kathryn, Ronneberger Olaf, Bates Russ, Žídek Augustin, Bridgland Alex, Alphafold 2. Fourteenth Critical Assessment of Techniques for Protein Structure Prediction, 2020.

[2] Baek Minkyung, DiMaio Frank, Anishchenko Ivan, Dauparas Justas, Ovchinnikov Sergey, Lee Gyu Rie, Wang Jue, Cong Qian, Kinch Lisa N, Schaeffer R. Dustin, Millán Claudia, Park Hahnbeom, Adams Carlos, Glassman Craig R, DeGiovanni Alexander, Pereira Jose H, Rodrigues Andrew V, van Dijk Amelie A, Ebrecht Andrea C, Opperman D. J., Sagmeister Tobias, Buhlheller Christoph, Pavkov-Keller Tea, Rathinaswamy Manoj K, Dalwadi Ujwala, Yip Christopher K, Burke John E, Garcia K. Christopher, Grishin Nick V, Adams Paul D, Read Randy J, and Baker David. Accurate prediction of protein structures and interactions using a three-track neural network. Science, 373(6557):871–876, 2021. doi: 10.1126/science.abj8754. URL https://www.science.org/doi/10.1126/science.abj8754. 34282049 PMC7612213

[3] Evans Richard, O’Neill Michael, Pritzel Alexander, Antropova Natasha, Senior Andrew, Green Timothy, Žídek Augustin, Bates Russell, Blackwell Sam, Yim Jason, Ronneberger Olaf, Bodenstein Sebastian, Zielinski Mirko, Bridgland Andrej, Potapenko Alexander, Cowie Charlie, Tunyasuvunakool Kathryn, Jain Ruchi, Clancy Ellen, Kohli Pushmeet, Jumper John, and Hassabis Demis. Protein complex prediction with alphafold-multimer. bioRxiv, page 2021.10.04.463034, 2022. doi: 10.1101/2021.10.04.463034. URL https://doi.org/10.1101/2021.10.04.463034.

[4] KrishnaRohith, WangJue, AhernWoody, SturmfelsPascal, VenkateshPreetham, KalvetIndrek, LeeGyu Rie, Morey-BurrowsFelix S, AnishchenkoIvan, HumphreysIan R, Generalized biomolecular modeling and design with rosettafold all-atom. Science, 384(6693): eadl2528, 2024.10.1126/science.adl252838452047

[5] Abramson Josh, Adler Jonas, Dunger Jack, Evans Richard, Green Tim, Pritzel Alexander, Ronneberger Olaf, Willmore Lindsay, Ballard Andrew J, Bambrick Joshua, Accurate structure prediction of biomolecular interactions with alphafold 3. Nature, pages 1–3, 2024. 10.1038/s41586-024-07487-w PMC11168924 38718835

[6] Discovery Chai, Boitreaud Jacques, Dent Jack, McPartlon Matthew, Meier Joshua, Reis Vinicius, Rogozhnikov Alex, and Wu Kevin. Chai-1: Decoding the molecular interactions of life. bioRxiv, pages 2024–10, 2024.

[7] Wohlwend Jeremy, Corso Gabriele, Passaro Saro, Reveiz Mateo, Leidal Ken, Swiderski Wojtek, Portnoi Tally, Chinn Itamar, Silterra Jacob, Jaakkola Tommi, Boltz-1 democratizing biomolecular interaction modeling. BioRxiv, 2024.

[8] Berman Helen M, Westbrook John, Feng Zukang, Gilliland Gary, Bhat Talapady N, Weissig Helge, Shindyalov Ilya N, and Bourne Philip E. The protein data bank. Nucleic acids research, 28(1):235–242, 2000. 10592235 10.1093/nar/28.1.235 PMC102472

[9] Geffner Tomas, Didi Kieran, Zhang Zuobai, Reidenbach Danny, Cao Zhonglin, Yim Jason, Geiger Mario, Dallago Christian, Kucukbenli Emine, Vahdat Arash, and Kreis Karsten. Proteina: Scaling flow-based protein structure generative models. 2025.

[10] Geffner Tomas, Didi Kieran, Cao Zhonglin, Reidenbach Danny, Zhang Zuobai, Dallago Christian, Kucukbenli Emine, Kreis Karsten, and Vahdat Arash. La-Proteina: Atomistic protein generation via partially latent flow matching. 2025.

[11] Qu Wei, Guan Jiawei, Ma Rui, Zhai Ke, Wu Weikun, and Wang Haobo. P(*all-atom*) is unlocking new path for protein design. August 2024.

[12] Yim Jason, Campbell Andrew, Foong Andrew YK, Gastegger Michael, Jiménez-Luna José, Lewis Sarah, Satorras Victor Garcia, Veeling Bastiaan S, Barzilay Regina, Jaakkola Tommi, Fast protein backbone generation with se (3) flow matching. arXiv preprint arXiv:2310.05297, 2023.

[13] Anishchenko Ivan, Kipnis Yakov, Kalvet Indrek, Zhou Guangfeng, Krishna Rohith, Pellock Samuel J, Lauko Anna, Lee Gyu Rie, An Linna, Dauparas Justas, Modeling protein-small molecule conformational ensembles with chemnet. bioRxiv, 2024. 10.1073/pnas.2427161122 PMC12625923 41187076

[14] Buttenschoen M., Morris G. M., and Deane C. M.. Posebusters: Ai-based docking methods fail to generate physically valid poses or generalise to novel sequences. arXiv preprint, 2023. URL http://arxiv.org/abs/2308.05777. 10.1039/d3sc04185a PMC10901501 38425520

[15] Rdkit: Open-source cheminformatics. https://www.rdkit.org. Accessed: 2025-08-12.

[16] Kunzmann Paul, Müller Thomas D., Greil Maximilian, Biotite: new tools for a versatile python bioinformatics library. BMC Bioinformatics, 24(1):236, 2023. doi: 10.1186/s12859-023-05345-6. URL https://doi.org/10.1186/s12859-023-05345-6. 37277726 PMC10243083

[17] Dauparas J, Anishchenko I, Bennett N, Bai H, Ragotte R J, Milles L F, Wicky B I M, Courbet A, de Haas R J, Bethel N, Leung P J Y, Huddy T F, Pellock S, Tischer D, Chan F, Koepnick B, Nguyen H, Kang A, Sankaran B, Bera A K, King N P, and Baker D. Robust deep learning–based protein sequence design using ProteinMPNN. Science, 378(6615):49–56, 2022 36108050 10.1126/science.add2187 PMC9997061

[18] Dauparas Justas, Lee Gyu Rie, Pecoraro Robert, An Linna, Anishchenko Ivan, Glasscock Cameron, and Baker David. Atomic context-conditioned protein sequence design using ligandmpnn. Nature Methods, pages 1–7, 2025. 40155723 10.1038/s41592-025-02626-1 PMC11978504

[19] Varadi M., Anyango S., Deshpande M., Nair S., Natassia C., Yordanova G., Yuan D., Stroe O., Wood G., Laydon A., Žídek A., Green T., Tunyasuvunakool K., Petersen S., Jumper J., Clancy E., Green R., Vora A., Lutfi M., Figurnov M., Cowie A., Hobbs N., Kohli P., Kleywegt G., Birney E., Hassabis D., and Velankar S.. Alphafold protein structure database: massively expanding the structural coverage of protein-sequence space with high-accuracy models. Nucleic Acids Research, 50:D439–D444, 2022. doi: 10.1093/nar/gkab1061. 34791371 PMC8728224

[20] Zhang Jing, Humphreys Ian R., Pei Jimin, Kim Jinuk, Choi Chulwon, Yuan Rongqing, Durham Jesse, Liu Siqi, Choi Hee-Jung, Baek Minkyung, Baker David, and Cong Qian. Computing the human interactome. bioRxiv, 2024. doi: 10.1101/2024.10.01.615885. URL https://doi.org/10.1101/2024.10.01.615885.

[21] Alford R. F., Leaver-Fay A., Jeliazkov J. R., O’Meara M. J., DiMaio F. P., Park H., Shapovalov M. V., Renfrew P. D., Mulligan V. K., Kappel K., Labonte J. W., Pacella M. S., Bonneau R., Bradley P., Dunbrack R. L. Jr, Das R., Baker D., Kuhlman B., Kortemme T., and Gray J. J.. The rosetta all-atom energy function for macromolecular modeling and design. Journal of Chemical Theory and Computation, 13:3031–3048, 2017. doi: 10.1021/acs.jctc.7b00125. 28430426 PMC5717763

[22] Bhardwaj Gaurav, O’Connor Jacob, Rettie Stephen, Huang Yen-Hua, Ramelot Theresa A, Mulligan Vikram Khipple, Alpkilic Gizem Gokce, Palmer Jonathan, Bera Asim K, Bick Matthew J, Di Piazza Maddalena, Li Xinting, Hosseinzadeh Parisa, Craven Timothy W, Tejero Roberto, Lauko Anna, Choi Ryan, Glynn Calina, Dong Linlin, Griffin Robert, van Voorhis Wesley C, Rodriguez Jose, Stewart Lance, Montelione Gaetano T, Craik David, and Baker David. Accurate de novo design of membrane-traversing macrocycles. Cell, 185(19):3520–3532.e26, September 2022. 36041435 10.1016/j.cell.2022.07.019 PMC9490236

[23] Hsu Chloe, Verkuil Robert, Liu Jason, Lin Zeming, Hie Brian, Sercu Tom, Lerer Adam, and Rives Alexander. Learning inverse folding from millions of predicted structures. In Chaudhuri Kamalika, Jegelka Stefanie, Song Le, Szepesvari Csaba, Niu Gang, and Sabato Sivan, editors, Proceedings of the 39th International Conference on Machine Learning, volume 162 of Proceedings of Machine Learning Research, pages 8946–8970. PMLR, 2022.

[24] The RNAcentral Consortium, Petrov Anton I, Kay Simon J E, Kalvari Ioanna, Howe Kevin L, Gray Kristian A, Bruford Elspeth A, Kersey Paul J, Cochrane Guy, Finn Robert D, Bateman Alex, Kozomara Ana, Griffiths-Jones Sam, Frankish Adam, Zwieb Christian W, Lau Britney Y, Williams Kelly P, Chan Patricia P, Lowe Todd M, Cannone Jamie J, Gutell Robin, Machnicka Magdalena A, Bujnicki Janusz M, Yoshihama Maki, Kenmochi Naoya, Chai Benli, Cole James R, Szymanski Maciej, Karlowski Wojciech M, Wood Valerie, Huala Eva, Berardini Tanya Z, Zhao Yi, Chen Runsheng, Zhu Weimin, Paraskevopoulou Maria D, Vlachos Ioannis S, Hatzigeorgiou Artemis G, Ma Lina, Zhang Zhang, Puetz Joern, Stadler Peter F, McDonald Daniel, Basu Siddhartha, Fey Petra, Engel Stacia R, Cherry J Michael, Volders Pieter-Jan, Mestdagh Pieter, Wower Jacek, Clark Michael B, Quek Xiu Cheng, and Dinger Marcel E. RNAcentral: a comprehensive database of non-coding RNA sequences. Nucleic Acids Res., 45(D1):D128–D134, January 2017. 27794554 10.1093/nar/gkw1008 PMC5210518

[25] Wayment-Steele Hannah K, Kladwang Wipapat, Strom Alexandra I, Lee Jeehyung, Treuille Adrien, Becka Alex, Participants Eterna, and Das Rhiju. RNA secondary structure packages evaluated and improved by high-throughput experiments. Nat. Methods, 19(10):1234–1242, October 2022. 36192461 10.1038/s41592-022-01605-0 PMC9839360

[26] Steinegger Martin and Söding Johannes. MMseqs2 enables sensitive protein sequence searching for the analysis of massive data sets. Nat. Biotechnol., 35(11):1026–1028, October 2017. 29035372 10.1038/nbt.3988

[27] Weirauch Matthew T., Yang Amy, Albu Mihai, Cote Adam G., Montenegro-Montero Anthony, Drewe Philipp, Najafabadi Hamed S., Lambert Samuel A., Mann Ivan, Cook Katherine, Zheng Hong, Goity Ariel, van Bakel Herman, Lozano Juan C., Galli Marco, Lewsey Matthew G., Huang Eric, Mukherjee Tapash, Chen Xue, Reece-Hoyes John S., Govindarajan Sridhar, Shaulsky Gad, Walhout Albertha J., Bouget François-Yves, Rätsch Gunnar, Larrondo Luis F., Ecker Joseph R., and Hughes Timothy R.. Determinants of DNA binding specificity of human transcription factors. Cell, 158(6):1431–1443, September 2014. ISSN 0092–8674. doi: 10.1016/j.cell.2014.08.009. 25215497 PMC4163041

[28] Siggers Trevor, Chang Albert B., Teixeira Andre, Wong Daniel, Williams Karen J., Ahmed Bashar, Ragoussis Jacques, Udalova Irina A., Smale Stephen T., and Bulyk Martha L.. Principles of dimer-specific gene regulation revealed by a comprehensive characterization of nf-*κ*b family dna binding. Nature Immunology, 2011. doi: 10.1038/ni.2151. (in press), Epub Nov 20, 2011. *Co-first authors: Siggers T, Chang AB. PMC3242931 22101729

[29] Hume Maxwell A, Barrera Luis A, Gisselbrecht Stephen S, and Bulyk Martha L. UniPROBE, update 2015: new tools and content for the online database of protein-binding microarray data on protein-DNA interactions. Nucleic Acids Res., 43(Database issue):D117–22, January 2015. 25378322 10.1093/nar/gku1045 PMC4383892

[30] Wingender E, Chen X, Hehl R, Karas H, Liebich I, Matys V, Meinhardt T, Prüss M, Reuter I, and Schacherer F. TRANSFAC: an integrated system for gene expression regulation. Nucleic Acids Res., 28(1):316–319, January 2000. 10592259 10.1093/nar/28.1.316 PMC102445

[31] Baek Minkyung, McHugh Ryan, Anishchenko Ivan, Baker David, and DiMaio Frank. Accurate prediction of nucleic acid and protein-nucleic acid complexes using rosettafoldna. bioRxiv, page 2022.09.09.507333, 2022. doi: 10.1101/2022.09.09.507333. URL https://doi.org/10.1101/2022.09.09.507333. PMC10776382 37996753

[32] Remmert Michael, Biegert Andreas, Hauser Andreas, and Söding Johannes. HHblits: lightning-fast iterative protein sequence searching by HMM-HMM alignment. Nat. Methods, 9(2):173–175, December 2011. 22198341 10.1038/nmeth.1818

[33] Kallenborn Felix, Chacon Alejandro, Hundt Christian, Sirelkhatim Hassan, Didi Kieran, Cha Sooyoung, Dallago Christian, Mirdita Milot, Schmidt Bertil, and Steinegger Martin. GPU-accelerated homology search with MMseqs2. November 2024. 10.1038/s41592-025-02819-8 PMC12510879 40968302

[34] Levine Daniel S, Shuaibi Muhammed, Spotte-Smith Evan Walter Clark, Taylor Michael G, Hasyim Muhammad R, Michel Kyle, Batatia Ilyes, Csányi Gábor, Dzamba Misko, Eastman Peter, Frey Nathan C, Fu Xiang, Gharakhanyan Vahe, Krishnapriyan Aditi S, Rackers Joshua A, Raja Sanjeev, Rizvi Ammar, Rosen Andrew S, Ulissi Zachary, Vargas Santiago, Zitnick C Lawrence, Blau Samuel M, and Wood Brandon M. The open molecules 2025 (OMol25) dataset, evaluations, and models. 2025.

[35] Mann Elias L, Wagen Corin C, Vandezande Jonathon E, Wagen Arien M, and Schneider Spencer C. Egret-1: Pretrained neural network potentials for efficient and accurate bioorganic simulation. 2025.

[36] Karras Tero, Aittala Miika, Aila Timo, and Laine Samuli. Elucidating the design space of diffusion-based generative models. arXiv preprint arXiv:2206.00364, 2022. Revised version v2 submitted on 11 Oct 2022; originally submitted 1 Jun 2022.

[37] Dehghani Mostafa, Djolonga Josip, Mustafa Basil, Padlewski Piotr, Heek Jonathan, Gilmer Justin, Steiner Andreas Peter, Caron Mathilde, Geirhos Robert, Alabdulmohsin Ibrahim, Jenatton Rodolphe, Beyer Lucas, Tschannen Michael, Arnab Anurag, Wang Xiao, Ruiz Carlos Riquelme, Minderer Matthias, Puigcerver Joan, Evci Utku, Kumar Manoj, Van Steenkiste Sjoerd, Elsayed Gamaleldin Fathy, Mahendran Aravindh, Yu Fisher, Oliver Avital, Huot Fantine, Bastings Jasmijn, Collier Mark, Gritsenko Alexey A., Birodkar Vighnesh, Vasconcelos Cristina, Tay Yi, Mensink Thomas, Kolesnikov Alexander, Pavetić Filip, Tran Dustin, Kipf Thomas, Lučić Mario, Zhai Xiaohua, Keysers Daniel, Harmsen Jeremiah J., and Houlsby Neil. Scaling vision transformers to 22 billion parameters. In Proceedings of the 40th International Conference on Machine Learning, volume 202 of Proceedings of Machine Learning Research, pages 7480–7512. PMLR, Jul 2023.

[38] Qiao Z., Nie W., Vahdat A., Miller III T. F., and Anandkumar A.. State-specific protein-ligand complex structure prediction with a multi-scale deep generative model. arXiv preprint, 2022

[39] Dunbar James and Deane Charlotte M. ANARCI: antigen receptor numbering and receptor classification. Bioinformatics, 32(2):298–300, January 2016. 26424857 10.1093/bioinformatics/btv552 PMC4708101

[40] Dunbar James, Krawczyk Konrad, Leem Jinwoo, Baker Terry, Fuchs Angelika, Georges Guy, Shi Jiye, and Deane Charlotte M. SAbDab: the structural antibody database. Nucleic Acids Res., 42(Database issue):D1140–6, January 2014. 24214988 10.1093/nar/gkt1043 PMC3965125

[41] Elliott Luc G, Simpkin Adam J, and Rigden Daniel J. ABCFold: easier running and comparison of AlphaFold 3, boltz-1 and chai-1. March 2025. 10.1093/bioadv/vbaf153 PMC12287924 40708869

